# Tuberculosis

**Published:** 1905-03

**Authors:** J. R. Briggs

**Affiliations:** House Physician to the Briggs-Clay Sanitarium for Consumptives, Dallas, Texas


### TUBERCULOSIS.

TUBERCLE—ETIOLOGY—HISTOGENESIS—CASEATION—MODES OF INFECTION—DIVERSE BEHAVIOR—PROPHYLAXIS—NORMAL IMMUNITY—ARTIFICIAL IMMUNITY — CULTURE PRODUCTS—THERAPEUTIC ACTION.

BY J. R. BRIGGS, M.D.,
House Physician to the Briggs-Clay Sanitarium for Consumptives, Dallas, Texas.

*Tubercle, Definition.*—Tubercle is a small granular non-vascular tumor or nodule formed within the body from new matter, resulting from the presence, in the tissues, of tubercle bacilli, with a tendency to purulent or cheesy degeneration and destruction of structure. Tubercles developed in various organs, or portions of the body, vary in size from a mere point to an eighth of an inch in diameter; when few in any organ they often pass into fibrous induration and remain harmless, but when numerous they form a tubercular mass that tends to destroy the surrounding structures, thus giving rise to consumption. No human, or organ, is immune against infection. This may all seem quite elementary, but this definition must be remembered amid all the multiplicity of phases presented, or we will often find ourselves at sea in the diverse manifestations of tubercle behavior. The infinitesimal tubercle here defined is the unit of the largest tubercular masses; a tubercular mass only one-half inch in diameter may contain many thousand perfectly formed tubercles.

*Etiology.*—This may be dismissed in few words; the definition alone gives the sole etiological factor in tuberculosis, in all its protean manifestations. Yet, much of the older teachings still seem to hold sway over the profession. "Heredity," "Discratia" and "Predisposition" still lift their heads against the only real source of germ infection. The old theories in this instance, as usual, die hard. Without tubercle bacilli we have no true tubercle.

*Histogenesis.*—The histological genesis of tubercle is best noted when injections of tubercle bacilli are made into the anterior chamber of the eye of animals. This being done, under strict antiseptic precautions, here is what follows: For the first few days after the inocculation no noticeable changes occur, there being only a rapid multiplication of bacilli in the aqueous humor, which soon begin to invade the structures of the iris, ciliary body, cornea, choroid and last the sclera—these tissues, however, remaining unaltered up to the sixth day. On the seventh day, or sometimes before, epithelioid cells begin to appear in the areas where the greater number of bacilli are aggregated; this is the initial step in the formation of tubercle. The bacilli now multiply more rapidly and are found in minute dense masses; a curious incident just here being the parallel arrangement of the germs. Karyokinetic figures become frequent, the proliferating process increasing with great rapidity, invades the fixed and connective tissue cells; later, the endothelial elements of the vessel walls and epithelial cell structures. By constant repetition of cell division large masses of epithelioid cells, rich in protoplasm are present. The bacilli are seen lying between the cells and frequently within them. Along with the epithelioid cells we see small round cells which are poor in protoplasm; these are the migratory cells which have escaped through the vessel walls and are, perhaps, influenced in their course by the lymph stream. Some investigators think these migratory cells carry the germs deeper into the surrounding structures. This process repeats itself in all situations in the body where the bacilli have invaded. After so long a time cell division ceases; the number of epithelioid cells is greatly increased in volume, many of which have two or three nuclei. Once in awhile, depending upon the character of the inocculation material, we note the presence of the giant cells of Langerhans, which contain many oval vesicular nuclei, in which are frequently found bacilli. In scrofula, in fact in all the more chronic forms of tuberculosis these giant cells are frequently found to be the only abode of the germs. In very acute forms, one giant cell may contain forty to sixty bacilli. When the nuclei form a complete circle the bacilli are found in its center; if, however, the nuclei are gathered at one pole the germs are usually found at the other extremity, thus showing a kind of antagonism between the nuclei and the bacilli. As the cellular proliferation decreases the leucocytic elements rapidly increase and within a period of ten or twelve days the inflammatory changes may be noted without magnification, as evidenced by the enlargement of the blood-vessels in the involved area. At this stage of capillary and venous distension the white corpuscles are seen to be against the walls of the vessels in a state of diapedesis. The epithelioid tubercle is more deeply and thoroughly filled with leucocytes, the number being constantly on the increase as the process continues. While the large-celled character of the tubercle is still seen, yet it slowly assumes the small-celled or lymphoid tubercle. Traumatism may render the histological changes so rapid as to cause it to represent the lymphoid charactar from start to finish. The bacilli having thus accomplished their mission we find either few or many very small gray translucent, cellular non-vascular nodules from the size of a pin's point up to the size of a millet seed and containing various numbers of germs.

*Caseation.*—Just here caseation, with its various retrograde changes, commences in the center of the tubercle and continues toward the periphery until there is complete death of all the tubercle cells. The leucocytes are first to show evidence of necrosis, then the epithelial cells; they, together with the fatty granules, present, form practically a homogeneous mass. Such tubercles look opaque or yellowish-white. The bacilli are still present and virulent, but later on also disintegrate as did the cells. The next step of involution is softening of the entire caseated areas and finally liquefaction, presenting the appearance of ordinary pus. Thus, tubercle ends and consumption begins.

*Modes of Infection.*—The infectious nature of tuberculosis is no longer questioned by any one. The recognition of the tubercle bacillus as the sole cause of tuberculosis lies as the basis of our modern attitude toward the subject. It only remains here to determine by what source and method the bacilli find ingress to the tissues and organs of the body. As simple as this problem may at first appear it is not yet solved. It is true many sources of infection are quite well established, but no authoritative source can yet demonstrate the most *frequent* method in human beings. At one time, and that quite recently, too, it seemed that the inhalation mode was practically the universal one; but when Behring, after long continued experiment on animals, concluded that the almost constant cause emanated from a bovine source all former theories were, in the minds of many, severely shaken. This great authority believes that most cases of tuberculosis come from infection in infancy and early childhood, by the use of milk from tuberculous cows; this he maintains after ascertaining that bovine tuberculosis is almost universal. He thinks that in such early period infections the disease may remain latent for many years. While this is not without reason, it is safer to rely upon the old theories of infection until the newer one rests upon the solid ground of demonstration. It will be a herculean task to prove that our greatest source of danger does not emanate from dried sputa. That this is quite a common means of contracting the disease I am quite positive. Of late I have given much attention to the lymph-glands as the initial seat of tuberculosis and have become convinced that from caseation of these glands the pulmonary or any other form of the disease may follow. If this be true and the secondary infection comes from the cervical glands it is quite plain that the bacilli were inhaled into the nose and mouth. It does not seem likely that the germs could lodge in the mucous folds, of adults, when being taken in food. Behring's theory, however, assumes that the bacilli pass into the soft succulent mucuous membrane where they find lodgment in the walls of the intestinal tract, from which they may be conveyed to other organs by either the lymphatic or vascular system. Some claim to have established the possibility of cutaneous infection. That the methods may be various there is no doubt. The essential thing to keep in mind is that the germ is not ubiquitous; it does not live and multiply outside of the body. Where infection occurs it must be from either animal or man with the disease. We must never lose sight of the fact, however, that we live, breathe and have our being in the midst of a tuberculous world. It is true all who are tubercular do not necessarily have consumption; the fact is but a small per cent. of tuberculous subjects ever develop phthisis, yet they carry in their bodies the virulent germ of tuberculosis and this is the exclusive potential factor in the dieease, which may be communicated in a multiplicity of ways.

*Diverse Behavior.*—We see this in the various forms of the disease from the most chronic conditions found in glands and bones up to the acute pneumonic type of the pulmonary manifestations and still further in the widely disseminated type of acute general miliary tuberculosis. Most of these gradations can be settled, as to origin, on clinical facts at the bedside. In the more chronic forms we find but few germs present in the tissues with perhaps a relative strong resistance. In the acute forms accidents and intercurrent diseases play a large rôle. In the acute general miliary form of tubercle blood vessels may rupture, or, old caseated areas may be disturbed, thereby flooding the vascular system with bacilli. Thus, after all, it is largely a question of dosage and weakened resistance, as to what course the disease may assume.

*Prophylaxis.*—Briefly, avoid the tubercle bacillus. This is, however, easier said than done. To keep away from the consumptive is to get out of the world; they are everywhere; no land is exempt and one country is about as free from it as another; the great alleged health resorts being the most dangerous places for habitation. The heavy responsibility of the profession lies in its duty to educate the public to a full recogniton of the dangers attending residence with the consumptive; and, where this can not be avoided the gospel of cleanliness and care in burning all sputa should be preached on all occasions. Until this is done the profession of medicine fails in its grand mission.

*Normal Immunity.*—That some races, families and individuals possess a stronger relative resistance against tuberculosis than others there can be no doubt. Of what this difference consists and the biological laws at its basis we do not yet know; and, I may add, we most likely shall never know. Immunity must reside primarily in protoplasm, but protoplasm is not an entity but a mechanism, and we do not know its intricate laws. This is about the sum total of all we know about the philosophy of normal, inherent immunity.

*Artificial Immunity.*—As to just what occurs in the production of artificially produced immunity we are equally ignorant. Just as in normal immunity, we know we have it, so in artificial immunity, we know we can produce it; and, that the latter can be as firmly established as the former, can be demonstrated to the full satisfaction of any one. I know of no literature on the protective inoculation of human beings against tuberculosis, except my own publications, the first of which appeared in 1901, the work having been done the year previous. If Behring can immunize cattle with the virulent germs, the animal manfacturing the tuberculin in its own body, why can not human beings be immunized by tuberculin manufactured in a laboratory? The toxins are surely the therapeutic agent in the bacilli. and what difference does it make, except as to safety to human beings, whether the toxins are extracted in a laboratory or in the body? I desire here, however, to speak more particularly with regard to the artificial production of immunity in human beings after the tubercular disease has made perceptible progress.

*Culture Products.*—The strangest thing to me, in this day and time, is the very remarkable attitude of indifference, and even skepticism, of the medical profession toward the culture products of the tubercle bacilli. That the tubercle bacilli contain a toxin which is curative in tuberculosis no well informed physican can for a moment doubt. Time and again this has been fully demonstrated, in fact, every day of all the passing years since Koch first announced his tuberculin in 1890. Still the very large majority of physicians yet maintain that there is no specific cure for tuberculosis; and, what is still more deplorable, they flatly refuse to investigate and make trials of the claims of those who are devoting their lives to the subject. I may truthfully go a step further and say that the work of those who have faithfully reported clinical results is questioned, indeed, emphatically denied! Who can account for this strange spirit of the profession? Are the members of the medical profession who annually report such a large per cent. of recoveries dishonest? Or, to put the matter in a more charitable light, is the curability of a large majority of all early stage cases of tuberculosis too good to be true?

As my line of work is devoted exclusively to the treatment of tuberculosis, and has been for many years, I feel it my duty to the profession to restate, briefly, the various steps of progress made in the research of this subject, as follows:

### TUBERCULIN, KOCH—FIRST ANNOUNCED BY PROF. R. KOCH, IN 1890.

*Mode of Preparation.*—Tubercle bacilli from pure culture are transferred into a flask containing sterile glycerine bouillon (1 per cent. beef extract, 1 per cent. pepton, 5 per cent. glycerine) and allowed to grow from six to eight weeks. The contents of the flask is then concentrated by boiling to one-tenth the original bulk, the tubercle bacilli filtered out, and the filtrate which now represents about 10 per cent. extract, 10 per cent. pepton, and 50 per cent. glycerine in water, is the original tuberculin proteids or toxins from the tubercle bacilli are present in small and varying amounts.

*Objections.*—Heat used, that is likely to injure the toxins; the uncertainty of the amount of toxins; the presence of beef extract and pepton, which latter cause malaise and fever when injected hypodermically, and thereby prevent the giving large enough doses of the toxin.

*Hunter's Modification of Koch's Tuberculin.*—Prepared by Hunter, of London, in 1891. The object sought was the removal of the objectionable beef extract and pepton and the glycerine. Process of preparing consists in precipitating Koch's Tuberculin with platinum chloride, which carries down albumoses and peptons. The filtrate is used.

*Objections.*—Heat used in the original tuberculin, that may injure the toxin; unknown quantity of toxins; their possible precipitation, with the reagent employed; the presence of more or less albumoses, extractives, and peptons from the original tuberculin.

*Klebs' Tuberculocidin.*—Prepared by Klebs, in 1893, from Koch's original tuberculin by precipitating the sodic iodide of bismuth, filtering, further precipitating the filtrate with absolute alcohol, collecting residue and redissolving in water one per cent. organic substances in solution.

*Objections.*—Heat used same as in all other preparations, unknown quantity of toxins, their possible precipitation by reagents employed, presence of albumose, meat extractives and pepton from the original tuberculin.

*Klebs' Antiphthisin.*—Prepared by Klebs, in 1894. Instead of using the original tuberculin, he used only the filtered glycerine bouillon upon which the tubercle bacilli had been grown, avoiding concentration by boiling heat and in subsequent steps, all degrees of heat that could be thought to injure the toxins, all other steps the same as in making tuberculocidin.

*Objections.*—Unknown quantity of pure toxins; their possible precipitation by reagents used; the presence of albumose, meat extractives and pepton from the glycerine bouillon.

*Tuberculinum Purificatum.*—Prepared by von Ruck, in 1896, by boiling in vacuum at 130 degrees F. the whole culture (tubercle bacilli and glycerine bouillon) for long periods (from four to six weeks) with the view of extracting more toxins from the specific germ; thereafter filtering out the tubercle bacilli, and proceeding the same as with tuberculocidin or antiphthisin.

*Objections.*—The same as to antiphthisin, more toxins were, however, present, the product being much more toxic than all other purifications.

*Koch's New "Tuberculin."*—Or Koch's Purified Tuberculin, announced by Koch, in April, 1897. Prepared from the bodies of tubercle bacilli, without using the fluid upon which they were grown; the tubercle bacilli were dried and powdered, and in that form put into water and transferred to the centrifugal machine, when after the fluid became clear in the middle portion, the top scum consising of fats and debris was drawn off, and only the clear part was decanted. To this was added enough glycerine to preserve it, one per cent. organic substance being present in the standardized solution.

*Objections.*—That the product can not be filtered through porcelain, and that it contains many tubercle bacilli, which by some experimenters were found to be virulent.

*Watery Extract of Tubercle Bacilli.*—Announced by von Ruck, in May, 1897. Prepared from the bodies of tubercle bacilli which are first washed, then dried and powdered. Then the fat of the tubercle bacilli (about thirty to thirty-five per cent.) is extracted with alcohol and ether; they are then dried and powdered again, and extracted in distilled water. This watery extract contains the pure toxins, passes readily through a porcelain filter, and the product is standardized to one one-hundredth, one-tenth and one per cent., being the three different solutions, No. 1, No. 10, and No. 100, respectively.

*Objections.*—None; the product being the pure toxin from the bacillus of tuberculosis.

*Serums.*—Aside from clinical trial of the above enumerated preparations (direct method) I have also given all the various serums (indirect method) a fair trial, and must say I have never, in a single instance, gotten any permanent clinical results from any of the serums. All they can contain is the transferred tuberculin, which has passed through the horse. So there is nothing in the various anti-tubercular serums, except the proprietorship and the money incidentally made by the manufacturers.

*Therapeutic Action of the Pure Toxins.*—I have treated, during the last five years, over 300 cases of tuberculosis with the pure toxin preparation—the Watery Extract of Tubercle Bacilli, von Ruck—and can, therefore, speak from actual experience, my work corroborating that of many others in the same line. Most of these cases were of the pulmonary form of tuberculosis, about 300; of the glandular variety, 47; of bones, 3; which are published elsewhere. Hence, I here only refer in detail to the pulmonary form as predominating in each case. I should here state that of this 300 cases of pulmonary tuberculosis, 240 were treated at the Sanitarium and 60 as out-patients. Cases comparatively clear of fever did quite well as out-patients; especially those of the better classes, who could afford the necessary rest and rich food. The lower classes, who were forced to labor during treatment, did poorly; yet, the per cent. of recoveries, even in such unfavorable cases, was a great surprise to me. The time required for the course of out-patients was much in excess of Sanitarium cases. Taking all the cases together, the report of recoveries was 70 per cent., but in the course of a year several (especially the poorer class) had recurrences which cut permanent results down to about 60 per cent. Other recurrences may perhaps occur, in the more recently restored, which may, in the course of years, make the permanent results as low as 55 per cent. Yet, even this, is, most assuredly, marvelous results in this well-nigh universally fatal disease.

Beginning on very small doses, hypodermically, which should be injected into the back and sides, the dosage is gradually increased until signs of reactions are noticeable; then the dose should be decreased or stopped altogether, this depending upon the degree of reaction. Thus each patient is given all the watery extract he can endure, short of local and systemic reaction, until there is full restoration to health and the disappearance of all tubercular deposits which the vascular supply can reach. The recurrent cases are those in whose lungs there are old encapsulated areas of caseated tubercle awaiting rupture from traumatism or intercurrent disease; such foci being inaccessible to vascular elements can not be absorbed.

There is immediate improvement of all symptoms from the very start. The appetite gets better, the digestion improves, the strength slowly returns, the cough grows less, though the expectoration is generally increased for the first few weeks, the annoying shortness of breath greatly improves, and the patient takes on from one-fourth to one pound of bodily weight daily until his former normal weight is reached. He feels himself again in a few weeks and has to be closely watched to keep him from injuring himself from over-exercise. His fever may, and often does, remain for many weeks and his cough, if he has secreting cavities, may not cease until long after restoration to good health—even months thereafter.

The average period of treatment in early-stage cases is about four months; they frequently, however, regain all their lost weight in from sixty to ninety days, and in many cases they weigh more on dismissal than ever before in life, even old men do this. The transformation which takes place in this short time is indeed most remarkable. I remember well the impression made on my mind when I discharged, as cured, my first cases, five years ago. They seemed to me to be quite well and said they so felt, but I could not believe the evidence of my own senses. Those first patients were in my mind day and night, for I was so sure they would relapse that I shunned them all I could; I did not want to meet them, so fearful was I that they would immediately go down again. But here they are, in this city, my very neighbors, and after five years are in perfect health. I retain, yet, the microscopic slides of these people's sputum and take them down occasionally and look at them. There are the millions of germs, as bright and fresh looking as if only prepared yesterday; and as I meditate, the man from whose lungs these bacilli come five years ago, passes me by and hollows "Hello, Doc!" Whether physicians believe what I here say oı not is a thing over which I have no control; yet, it is plainly my duty to tell it and let results take care of themselves.

Just how the extract of tubercle baccilli cures tuberculosis I do not know. Neither do I know how the mixed treatment cures syphilis; yet this ignorance of the modus operandi would not prevent me from using the treatment in appropriate cases. That the bacilli toxins will disintegrate tubercle I know to be a fact; for I have seen this occur under my very eyes. How it does it is not known and perhape may never be; yet, I have my own views on the subject, but this is no place for speculation. I will say, however, that it is my conviction that the primary therapeutic action resides in cell stimulation with greatly increased metabolism, which, if pushed too fast, eventuates in local reactions in the tubercular area. It seems to have also a secondary or local action on the tubercle cell, which I regard as truly specific, thereby disintegrating large tubercular masses. In glandular tuberculosis where rapidly increasing doses are administered quite violent local reactions occur; and, in cases of bone and joint tuberculosis I have seen the inflammation so great from large doses as to simulate acute inflammatory rheumatism. Its action may be perfectly seen in the larynx, eye and other locations. Wherever tubercle is found it reacts under this toxin and with the repetition of such reactions the tubercular mass is completely broken up and absorbed.

2

I here refer to live tubercle only and have no reference to necrossed tissues upon which no remedy is, for once, supposed to act.

For several years past many good and true physicians have published results similar to the above, but they were simply winked at and passed by. It is to be hoped that physicians will wake up to a realization of their obligations to their patients and see also that they are sleeping on their own rights. Pulmonary tuberculosis, in its earlier stages, is the most easily curable of any of the grave forms of diseases and the physician who does not realize this is reprehensible to his clientele.

*Sanitarium Place.*

